# Genomic and transcriptomic analyses of the tangerine pathotype of *Alternaria alternata* in response to oxidative stress

**DOI:** 10.1038/srep32437

**Published:** 2016-09-01

**Authors:** Mingshuang Wang, Xuepeng Sun, Dongliang Yu, Jianping Xu, Kuangren Chung, Hongye Li

**Affiliations:** 1 Institute of Biotechnology, Zhejiang University, Hangzhou 310058, China; 2 Boyce Thompson Institute, Cornell University, Ithaca 14850, USA; 3 College of Life and Environmental Sciences, Hangzhou Normal University, Hangzhou 310018, China; 4 Department of Biology, McMaster University, Hamilton, Ontario L8S 4K1, Canada; 5 Department of Plant Pathology, National Chung-Hsing University, Taichung 40227, Taiwan

## Abstract

The tangerine pathotype of *Alternaria alternata* produces the *A*. *citri* toxin (ACT) and is the causal agent of citrus brown spot that results in significant yield losses worldwide. Both the production of ACT and the ability to detoxify reactive oxygen species (ROS) are required for *A*. *alternata* pathogenicity in citrus. In this study, we report the 34.41 Mb genome sequence of strain Z7 of the tangerine pathotype of *A*. *alternata*. The host selective ACT gene cluster in strain Z7 was identified, which included 25 genes with 19 of them not reported previously. Of these, 10 genes were present only in the tangerine pathotype, representing the most likely candidate genes for this pathotype specialization. A transcriptome analysis of the global effects of H_2_O_2_ on gene expression revealed 1108 up-regulated and 498 down-regulated genes. Expressions of those genes encoding catalase, peroxiredoxin, thioredoxin and glutathione were highly induced. Genes encoding several protein families including kinases, transcription factors, transporters, cytochrome P450, ubiquitin and heat shock proteins were found associated with adaptation to oxidative stress. Our data not only revealed the molecular basis of ACT biosynthesis but also provided new insights into the potential pathways that the phytopathogen *A*. *alternata* copes with oxidative stress.

*Alternaria alternata* is ubiquitously distributed in air, soil and various decaying plant materials^1^. Strains of this species can cause diseases on plants resulting in significant crop losses worldwide. Host-selective toxins (HSTs) are the essential pathogenic factors for virulent *A*. *alternata*. At least seven HSTs produced by *A*. *alternata* have been recognized, with each showing a specific toxicity to one host plant species, including the Japanese pear, strawberry, tangerine, apple, tomato, rough lemon and tobacco^2^. These HST-producing *A*. *alternata* pathogens produce conidia with similar morphologies and can be distinguished only based on their host preferences^3^. Thus, according to the characters and host range of HSTs, HST-producing *A*. *alternata* are usually assigned to seven pathotypes. Except for the tobacco pathotype, HSTs differing in chemical structures have been purified from other six *A*. *alternata* pathotypes. ACT produced by the tangerine pathotype, *A*. *fries* toxin (AFT) by the strawberry pathotype and *A*. *kikuchiana* toxin (AKT) by the Japanese pear pathotype share a 9,10-epoxy-8-hydroxy-9-methyl-decatrienoic acid (EDA) core moiety^4^,^5^,^6^. Genes required for EDA formation are organized in a similar manner among the three *A*. *alternata* pathotypes, while the compositions of other genes resided in the cluster are very different^2^.

The tangerine pathotype of *A*. *alternata* produces a host-selective ACT. Seven genes, *ACTT2*, *ACTT3*, *ACTTR*, *ACTT5*, *ACTT6*, *ACTTS2* and *ACTTS3* are required for the biosynthesis of ACT. RNA silencing or disruption of these genes led to the loss of ACT production and pathogenicity^7^,^8^,^9^,^10^,^11^. However, whether there are genes that are unique to the tangerine pathotype and if there are other additional candidate genes involved in the biosynthesis and regulation of ACT remain to be investigated.

In general, plants cells can rapidly generate large amount ROS in an oxidative burst as a defense response in the early events of plant-microbe interactions^12^. High ROS levels can cause a series of molecular damage such as DNA mutations, protein misfolding, and lipid peroxidation, which can eventually lead to metabolic dysfunction and cell death^13^. To cope with the oxidative stress and colonize host plants, plant pathogens have evolved many strategies to neutralize ROS. Both enzymatic and non-enzymatic systems involving superoxide dismutase, peroxidases and glutathione, can scavenge intracellular toxic ROS^14^. The mitogen-activated protein kinase Hog1, a common stress response regulator with well characterized functions in response to hyperosmolality, has been found to be essential for oxidative stress resistance in *Aspergillus fumigatus*, *Botryotinia fuckeliana* and *Cochliobolus heterostrophus* (Du, Sarfati *et al*. 2006; Segmuller, Ellendorf *et al*. 2007; Igbaria, Lev *et al*. 2008). In *B*. *cinerea*, *Ustilago maydis* and *Magnaporthe oryzae*, the bZIP transcription factorYap1 was found to be the main regulator that mediates ROS detoxification (Molina and Kahmann 2007; Temme and Tudzynski 2009; Guo, Chen *et al*. 2011).

Recently, several outstanding studies have provided novel insights into the mechanisms for cellular protection against the toxicity of host ROS involved in *A*. *alternata*. It has been known that apart from HST, the ability to alleviate ROS by the tangerine pathotype of *A*. *alternata* is also crucial for pathogenesis to citrus^15^,^16^,^17^,^18^. Several genes which encode different kinds of proteins including the redox-responsive Yap1-like transcription factor, the Skn7 response regulator, the Hog1 MAP kinase, the Nox NADPH oxidases, the Nps6 non-ribosomal peptide synthetase, and the Gpx3 glutathione peroxidase, have shown to be required for ROS detoxification and full virulence on citrus^15^,^16^,^17^,^18^,^19^. However, these findings were established only through the functional analysis and gene expression profiling in mutant strains^15^,^16^,^17^,^18^ and the interrelationships among these genes have not been established. In addition, the mechanisms responsible for other stresses, and the genes involved in sporulation, which is an essential characteristic of the disease cycle of citrus brown spot, are completely unknown. For these reasons, we have fully sequenced the genome of a tangerine pathotype strain of *A*. *alternata* and performed a comparative genomics analysis. Furthermore, we carried out global transcriptome analysis of this fungus after H_2_O_2_ treatment to investigate the genes that are differentially expressed to help identifying the potential genes and metabolic pathways by which the fungus uses to cope with oxidative stress.

## Results and Discussion

### General features

**Figure 1:**
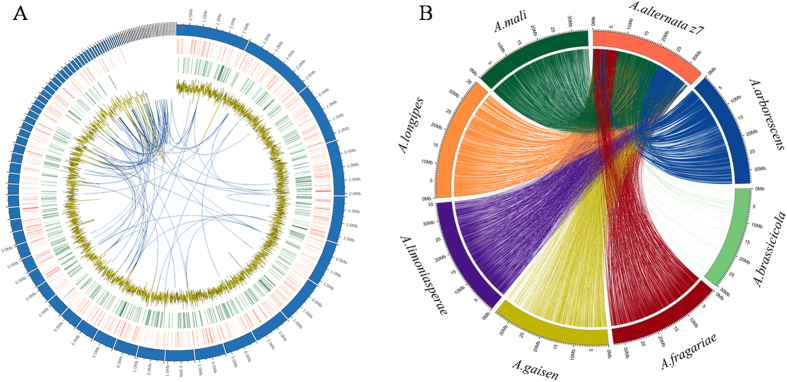
Genome sequence analysis. (**A**) Genome organization and gene distribution in *A*. *alternata* Z7. The peripheral circle represents 103 contigs each with a size of over 5 Kb. The second and third circles of color bands show the genes that were up-regulated and down-regulated after H_2_O_2_ treatment, respectively. Higher intensity of the color represents a larger log2 fold change of gene expression. The fourth circle shows the GC content in 10 Kb windows with a step of 2 Kb. Gene duplications are shown in the center. (**B**) Genomic synteny of *A*. *alternata* Z7 with other 7 *Alternaria* species. The identity and length cutoff were set at 80% and 10 kb, respectively.

The genome assembly of *A*. *alternata* strain Z7 was constructed using a combination of Illumina and Pacbio reads. The final assembly included 161 contigs (>1000 bp) with a total genome size of 34.41 Mb (Fig. 1A, Table 1). The genome size of *A*. *alternata* Z7 was approximately 11% larger than that of *A*. *brassicicola*, 25% smaller than that of *A*. *destruens*, and comparable with other *Alternaria* species (Supplementary Table S1). The gene density of strain Z7 is similar to those of other sequenced *Alternaria* species, at ~351 genes per Mb. Among the sequenced *Alternaria* spp., *A*. *solani* has the highest density (377 genes per Mb) and *A*. *destruens* has the lowest (271 genes per Mb) (Supplementary Table S1). Large-scale genome synteny was found between *A*. *alternata* Z7 and the other *Alternaria* species with the exception of *A*. *brassicicola* (Fig. 1B). An orthoMCL analysis identified 11611 orthologous groups (containing 11660 proteins) in these seven pathotypes of *Alternaria*, many more than those between *A*. *alternata* Z7 and *A*. *brassicicola* (8180 proteins in 8003 orthologous groups). These results revealed a high degree of genome similarity across *Alternaria* strains living in different ecological niches and/or with different hosts specificities.

### Phylogenetic analysis

**Figure 2:**
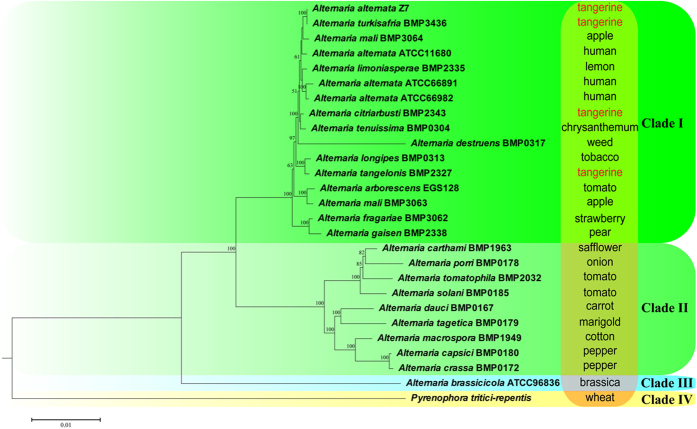
Phylogenomic relationships of *A*. *alternata* Z7 with other fungi. The maximum likelihood (ML) phylogenetic tree was built with the program MEGA6 using the Jones-Taylor-Thornton (JTT) amino acid substitution model. The corresponding host for each species was listed in the right column. Node supported as ML bootstraps (values ≥50%) are displayed above or below each branch.

A phylogenetic tree based on a combined analysis of 200 conserved single-copy orthologs randomly selected from 26 *Alternaria* species and *Pyrenophora tritici-repentis* was constructed and revealed highly close interspecific genetic relationships of the distinct pathotypes of *A*. *alternata* (Fig. 2). The systematics of *Alternaria* has been ambiguous. Simmons had assigned 77 *Alternaria* isolates from citrus into 10 species according to sporulation patterns^20^. However, researchers failed to delineate significant variation among those species based on DNA sequences of popular marker genes, such as those coding for calmodulin, translation elongation factor alpha, chitin synthase and 1, 3, 8-trihydroxynaphthalene reductase and actin^21^. Indeed, based on ITS, the small-spored, HST-producing *A*. *alternata* pathotypes of tangerine, rough lemon, strawberry, tomato, apple, and pear could not be differentiated from each other or from several saprophytic isolates of *A*. *alternata*^22^. To help reduce taxonomic confusions, several sections within the genus *Alternaria* have been proposed^23^,^24^,^25^. In our phylogenetic tree, three clades Clade I, Clade II and Clade III corresponded well with the proposed section ‘*Alternaria*’, ‘*Porri*’ and ‘*Brassicicola*’, providing powerful support for the new systematics of *Alternaria*^23^. Still, many of the morphospecies within section *Alternaria* cannot be distinguished, even with sequence information from 200 marker genes. *A*. *alternata* Z7, *A*. *citriarbusti*, *A*. *tangelonis* can all produce ACT and cause citrus brown spot; however, they were grouped into separate branches in the phylogenetic tree. A similar situation was found with the apple pathotype species, for which *A*. *mali* BMP3063 and *A*. *mali* BMP3064 were grouped into different branches. Thus, phylogenetic relationships of *Alternaria* species are not completely correlated with their host ranges. Indeed, host specificity of the *A*. *alternata* isolates is determined by the HST which is encoded by a single cluster in the pathotypes and this cluster resides on the conditionally dispensable (CD) chromosomes^2^. By sequencing the CD chromosomes in three pathotype of *A*. *alternata*, one recent study identified large syntenic regions among the three CD chromosomes^26^. However, the HST clusters were unique to the respective pathotypes^26^. In contrast, *A*. *alternata* Z7 and *A*. *turkisafria* can both infect citrus and were clustered together in the phylogenetic tree, suggesting that they have speciated recently (Fig. 2).

### Unique genes in the tangerine pathotype

**Figure 3:**
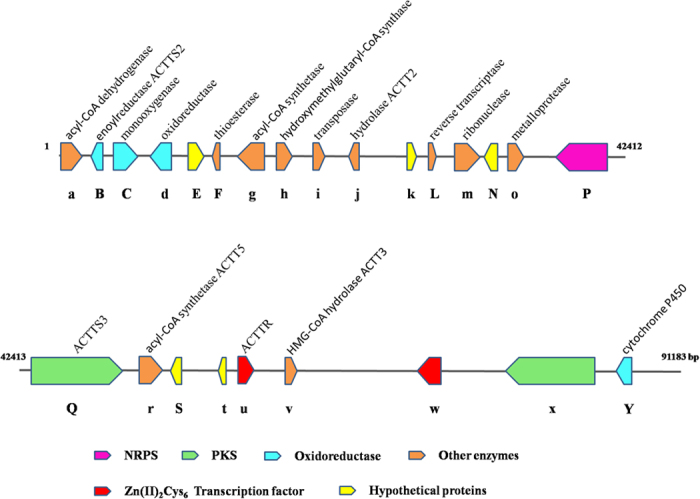
Characterization of the host specific ACT gene cluster in *A*. *alternata* Z7. The ACT gene cluster was located in a ~91.2 kb contig containing 25 genes. Capital letters represent genes uniquely present in the tangerine pathotype of *A*. *alternata*.

As described above, although they have distinct host range, the small-spored, HTS-producing *A*. *alternata* could not be distinguished even with sequences from 200 genes. We wondered which genes represent the specific determinants for *A*. *alternata* to attack tangerines and the hybrids of tangerine and orange. To answer this question, we searched all the orthologous groups in the genomes of 26 *Alternaria* species. Those that were only present in all 4 tangerine pathotype strains and with their identities to each other over 80% were retrieved. Ten genes were identified to be uniquely present in the tangerine pathotype (Supplementary Table S2). These genes were predicted to encode several types of enzymes, such as enoylreductase, monooxygenase, thioesterase, reverse transcriptase, non-ribosomal peptide synthetase and polyketide synthase. Three of the ten genes encode hypothetical proteins. Intriguingly, these genes were found to be clustered in the Z7 genome. Subsequent analysis of the secondary metabolite biosynthetic gene clusters confirmed that they are important members of the ACT gene cluster (Fig. 3 and Supplementary Table S5).

### ACT gene cluster

A total of 12 polyketide synthases (PKSs), 7 non-ribosomal peptide synthetases (NRPSs), 1 PKS-like and 5 NRPS-like genes were identified in the genome of *A*. *alternata* Z7. These genes have been found to be responsible for the biosynthesis of the backbone structure of many secondary metabolites. The number of backbone genes in strain Z7 is similar to that in *Nectria haematococca* but much smaller than those in other fungi, such as *Magnaporthe grisea* and *Fusarium graminearum* (Supplementary Table S3). The backbone genes in Z7 are organized into 18 secondary metabolite biosynthetic gene clusters or partial clusters. So far, several secondary metabolites such as alternariol, alternariol-9-methyl ether, dimethyl coprogen, alternapyrone and HSTs have been successfully identified^2^,^27^,^28^,^29^. However, the exact products of most of the clusters in Z7 remain largely unknown (Supplementary Table S4). The host selective ACT gene cluster in Z7 was found to be located in a 91.2 kb DNA fragment containing 25 genes and most of them have 2 or 3 copies, indicating the strong capacity to synthesize the ACT (Fig. 3). Proteins found in the ACT biosynthetic cluster include polyketide synthase PKS, NRPS, enoyl-CoA hydratase, HMG-CoA hydrolase, acyl-CoA dehydrogenases, cytochrome P450 monooxygenase, and transcription factor (Fig. 3 and Supplementary Table S5). Previously reported genes, *ACTT2*, *ACTT3*, *ACTTR*,*ACTT5*, *ACTTS2* and *ACTTS3*, were found in the cluster, while *ACTT6* was not included in this cluster but was found in another contig (3503 bp in length), indicating the gene cluster responsible for ACT biosynthesis may be larger. These toxin-related genes except for AALTg11737, AALTg11744 and AALTg11742 also were found in *A*. *citriarbusti*, *A*. *tangelonis* and *A*. *turkisafria*. Both AALTg11737 and AALTg11744 are present only in Z7, and AALTg11742 is present in Z7 and *A*. *citriarbusti* only (Supplementary Table S5). All the above-mentioned 10 tangerine pathotype unique genes are included in this gene cluster, two of them have been previously reported as unique to the tangerine pathotype, i.e., enoylreductase ACTTS2 and polyketide synthase ACTTS3^10^,^11^. Whether the remaining 8 specific genes play a critical role in ACT biosynthesis needs to be functionally elucidated.

### Carbohydrate-active enzymes and secretomes

**Figure 4:**
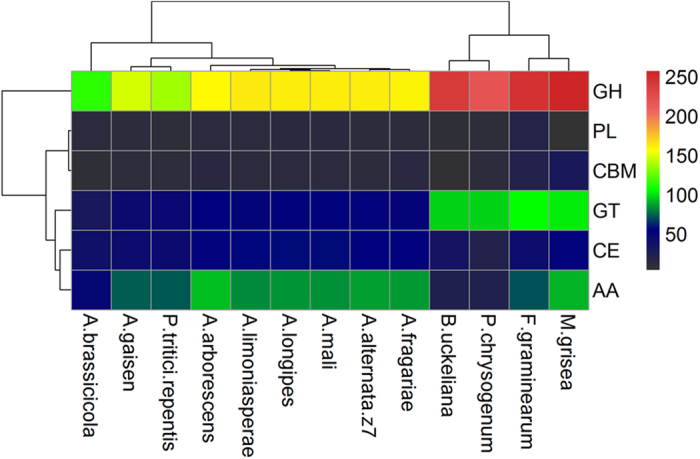
Distribution of CAZymes among different fungi. The number of CAZymes in each main class is shown by a color gradient. GH: Glycoside Hydrolase, PL: Polysaccharide Lyase, CBM: Carbohydrate-Binding Modules, GT: Glycosyl Transferase, CE: Carbohydrate Esterases, AA: Auxiliary Activities.

Carbohydrate-active enzymes (CAZymes) are responsible for the degradation of glycol-conjugated and oligo- and polysaccharides. They play important roles in the acquisition of nutrients from the environment for fungi. A total of 373 putative CAZyme genes were identified in Z7. These include 51 GlycosylTransferases (GTs), 161 Glycoside Hydrolases (GHs), 53 Carbohydrate Esterases (CEs), 11 Polysaccharide Lyases (PLs), 86 Auxiliary Activities (AAs) and 11 Carbohydrate-Binding Modules (CBMs). The types and numbers of CAZymes among different pathotypes of *A*. *alternata* are similar; however, *A*. *gaisen* has fewer CAZymes, i.e., 328 in *A*. *gaisen* versus 372~381 in other pathotypes (Fig. 4). *A*. *alternata* Z7 (53) has more CE than *B*. *fuckeliana* (35), *Penicillium chrysogenum* (21), *F*. *graminearum* (42) and *M*. *grisea* (51) but has fewer GH (161 versus 219~257) and GT (51 versus 97~106) than them (Fig. 4). Those different enzymes are mostly related to the degradation of plant cell wall components. Compared to *B*. *fuckeliana*, *F*. *graminearum* and *M*. *grisea*, Z7 has fewer (6 versus 17~24) xyloglucan transferases (GH16) responsible for degrading xylan and fewer (11 versus 16~21 and 7 versus 13~16) multifunctional catabolic enzymes (GH3, GH5) involved in the decomposition of plant pectin and hemicellulose^30^,^31^. Z7 lacks the α-glucuronidases (GH115) which is only active on xylan oligomers in ascomycetous fungi^32^ (Supplementary Table S6). However, Z7 has relatively high numbers of the AA3 family of cellobiose dehydrogenase (27), the AA7 family of glucooligosaccharide oxidase (17) and the AA9 (formerly GH61) family of polysaccharide monooxygenases (23) involved in degrading cellulose^31^,^33^ (Supplementary Table S6). The differences in the composition of CAZymes could help identify the CAZymes required for citrus infection in *A*. *alternata* Z7.

### Identification of differentially expressed genes (DEGs)

Since ROS-detoxification is vital for *A*. *alternata* survival within plant hosts, cellular responses to oxidative stress are necessarily required for pathogenicity. To analyze the transcriptional response of *A*. *alternata* Z7 to H_2_O_2_, mycelia were treated with 15 mM H_2_O_2_ and samples were collected 30 minutes later. In total, we identified 1606 differentially expressed genes at 30 min post-treatment, 1108 of them displayed increased transcript levels and 498 of them displayed decreased transcript levels (Fig. 1A). To validate the DEG data, transcript levels of eighteen selected genes that showed different expression patterns in response to the H_2_O_2_ treatments were confirmed using quantitative reverse transcription PCR (qRT-PCR). While the magnitude of fold changes differed between the two methods for some of the genes, all 18 tested genes showed similar trends in transcript accumulation between the transcriptome and qRT-PCR results (Supplementary Fig. S2). These results showed that our DEG data was reliable for further analysis.

### Functional analysis of DEGs

**Figure 5:**
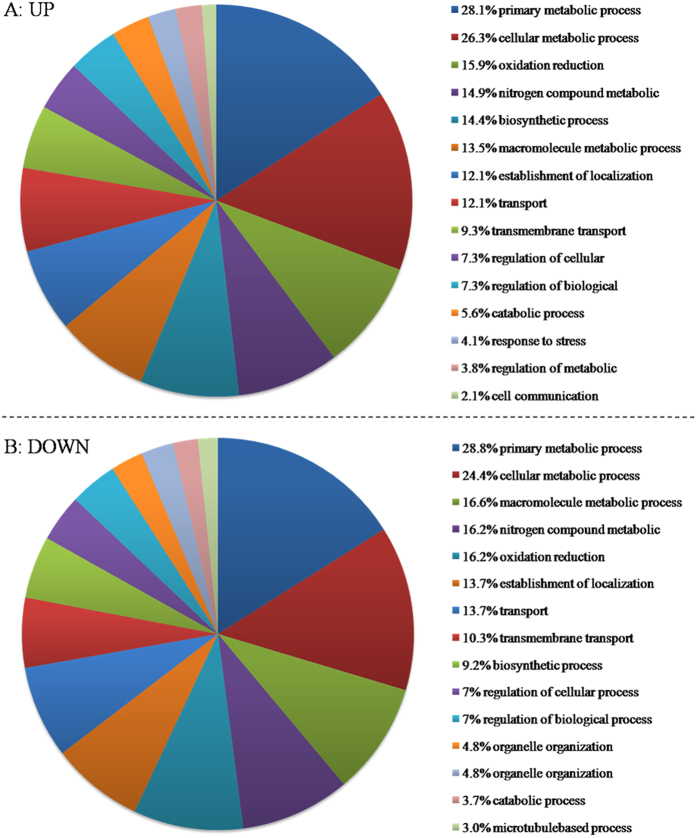
The percentage of *A*. *alternata* transcripts belonging to each GO Slim term for the secondary GO category of biological processes. (**A**) The enrichment of the up-regulated genes. (**B**) The enrichment of the down-regulated genes. Only the15 most frequent GO Slim terms are shown.

Of the differentially expressed genes, 659 up-regulated and 271 down-regulated genes could be assigned into functional categories of the gene ontology (GO): biological process, molecular function, and cellular component. GO terms predominantly enriched in both the up-regulated and down-regulated genes were involved in the following biological processes: ‘primary metabolic process’ (GO:0044238), ‘cellular metabolic process’ (GO:0044237),‘oxidoreductase activity’ (GO:0016491), ‘nitrogen compound metabolic process’ (GO:0006807),‘macromolecule metabolic process’ (GO:0043170), ‘establishment of localization’ (GO:0051234) and ‘transport’ (GO:0006810) (Fig. 5). Moreover, the category ‘biosynthetic process’ (GO:0009058) was also over-represented for the up-regulated genes (Fig. 5). There were 149 genes exhibiting differential expression in the category ‘oxidoreductase activity’, with105 being induced and 44 being repressed under oxidative stress. These genes belonged to a diversity of dehydrogenases and oxidases and they mainly participate in primary metabolic processes like amino acid synthesis and degradation, glucose and lipid metabolism (Supplementary Table S7). The three most up-regulated genes in this category encoded 2,4-dichlorophenol 6-monooxygenase (AALTg3456, log2FC 5.1), alcohol dehydrogenase (AALTg3691, log2FC 4.7) and glutathione reductase (AALTg2809, log2FC 3.3). The three enzymes with the most down-regulation were uricase (AALTg5212, log2FC -2.56), nitrite reductase (AALTg4186, log2FC -3.31) and C-5 sterol desaturase (AALTg6701, log2FC -3.53).

### Differential expression of antioxidation genes

ROS homeostasis in most organisms is maintained through the balance between ROS production and ROS scavenging. Most organisms have evolved oxidative stress response mechanisms to scavenge elevated intracellular ROS levels by antioxidant enzymes. Those enzymes include catalase, glutathione peroxidase, ascorbate peroxidase and superoxide dismutase, which are known as universal antioxidant enzymes involved in ROS detoxification in all living organisms. We examined the expression of genes encoding those scavengers. Transcript levels of 12 peroxidases were significantly up-regulated after H_2_O_2_ treatment, including three catalases, one catalase-peroxidase, four cysteine peroxiredoxin, one ascorbate peroxidase, one glutathione peroxidase (AaGpx3), one carboxymuconolactone decarboxylase and one hybrid ascorbate-cytochrome C peroxidase (Table 2). Our result is consistent with the recent finding that glutathione peroxidase AaGpx3 in *A*. *alternata* was essential for the detoxification of cellular stresses induced by ROS^18^. Notably, three Fe-Mn type superoxide dismutases and four Fe-Cu type superoxide dismutases were identified in the *A*. *alternata* Z7 genome, however, none of them showed significantly different expression during H_2_O_2_ stress.

Non-enzymatic defense systems against ROS include compounds that are oxidized by ROS and thereby reduce oxidant levels in cells^34^. Thioredoxin is a class of small proteins that act as electron donors to ribonucleotide reductases and peroxidases^35^. We examined the expression of all the thioredoxin encoding genes and found that seven of them were significantly up-regulated (Table 2). Thioredoxins in *Cryptococcus neoformans* and *Ascochyta rabiei* are also known to be associated with oxidative stress tolerance, suggesting the conserved role thioredoxin played in fungi^36^,^37^. Glutathione is a thiol-containing tripeptide which maintains the intracellular redox homeostasis by reducing cellular disulfide bonds^38^. Besides the *AaGpx3*, 10 other genes involved in glutathione metabolism were also highly induced (Table 2). It is worth mentioning that no gene in this pathway was down-regulated in transcript abundance. The enrichment of up-regulated genes in this pathway suggests that the glutathione system plays an important role in the elimination of ROS in *A*. *alternata*.

### Kinases

Protein kinases are responsible for the phosphorylation of proteins and participate in various cell processes. A total of 137 kinases were identified in the genome of *A*. *alternata* Z7. Among these, the transcript levels of 10 kinases were significantly elevated and six were down-regulated during H_2_O_2_ stress (Supplementary Table S8). In some fungi including *A*. *alternata*, the transcript levels of the oxidative stress response genes are controlled by the mitogen-activated protein kinase (MAPK) Hog1^39^,^40^,^41^. In our data, the expression of *AaHog1* (AALTg10096, log2FC 1.2) genes in *A*. *alternata* Z7 were significantly up-regulated during H_2_O_2_ treatment, confirming its important role in ROS scavenging. Moreover, several genes involved in the Hog1 MAPK signaling pathway were also up-regulated in their expression. These include the histidine phosphotransfer protein Ypd1 involved in the osmolarity two-component sensing and response system (AALTg9320, log2FC 1.1), protein phosphatase Ptc1 (AALTg9981, log2FC 1.1) and tyrosine-protein phosphatase Ptp2 (AALTg5082, log2FC 2.2). These genes are known to be associated with development, stress response, signal transduction and virulence at varying degrees in different fungal species^42^,^43^,^44^. However, there has been little evidence that these genes are involved in ROS elimination. Another differentially expressed kinaseis the sucrose non-fermenting protein Snf1, which is a serine/threonine protein kinase and plays a key role in controlling carbon source utilization^45^,^46^. The Snf1 protein kinase is also involved in regulating a broad range of cellular and morphogenetic processes, such as spore formation, filamentation and invasive growth, autophagy, virulence, as well as response to environmental stresses including oxidative stress, heat shock and alkaline pH^46^,^47^,^48^,^49^,^50^. Induced expression of this important kinase (AALTg3740, log2FC 1.3) was observed in our transcriptome data, suggesting a potential role of Snf1 in ROS-detoxification in *A*. *alternata*.

### Transcription factors

The expression of the oxidative stress response genes can also be controlled by distinct transcription factors. For example, homologs of the yeast YAP1-like transcription factor are the main regulators of ROS resistance in many filamentous fungi^19^,^51^,^52^. The *A*. *alternata* Z7 genome contains 283 transcription factors belonging to 19 subfamilies. The largest subfamily is theZn_2_/Cys_6_, which includes 147 members while the second largest is the C_2_H_2_ zinc-finger subfamily with 47 genes. These two subfamilies account for about 70 percent of the total transcription factors in *A*. *alternata* Z7 (Supplementary Table S9). After exposure of the *A*. *alternata* strain Z7 to H_2_O_2_ for 30 min, 33 differentially expressed transcription factors were discovered with the expressions of 19 being up-regulated and 14 being down-regulated, respectively (Supplementary Table S10). The transcript level of the *AaYap1* gene (AALTg912, log2FC 1.5) was significantly induced as was expected. However, the heat shock factor type DNA-binding transcription factor AaSkn7 (AALTg8622), which was recently revealed to be involved in cellular resistance to oxidative stress and pathogenicity to citrus in *A*. *alternata*, did not show significantly different expression between the two treatments in our investigation^53^. The results suggest that *AaSkn7* is essential but not specifically involved in response to H_2_O_2_ stress. Interestingly, we discovered that the transcript level of the nitrate-specific transcription factor *NirA* homolog (AALTg8635, log2FC, 1.3) was apparently increased after H_2_O_2_ treatment. NirA activates the expression of the nitrate assimilation genes when nitrate or nitrite is present^54^. Most of the remaining transcription factors belong to the subfamilies of Zn_clus, zf-C_2_H_2_, Myb_DNA-binding and HLH and the functions of their encoding genes are largely unknown in oxidative stress response (Supplementary Table S10).

### MFS and ABC transporters

The major facilitator superfamily (MFS) and the ATP-binding cassette (ABC) transporters are the top two biggest classes of transporters in fungi. Members of the former mainly play roles in nutrient uptake and drug efflux while the latter transport a broad range of compounds like ions, drugs and sugars^55^. Involvement of these two families of transporters in multidrug resistance (MDR) has been widely investigated. The MDR transporter ABC3 in *M*. *grisea* was also revealed to play an important role in pathogenesis and in response to intracellular oxidative stress^56^. After exposure of *A*. *alternata* Z7 to H_2_O_2_, 28 and 14 genes encoding MFS were significantly induced and repressed, respectively, while the expressions of seven and one ABC transporter genes were up-regulated and down-regulated, respectively (Supplementary Table S11). Of the 28 up-regulated MFS genes in *A*. *alternata* Z7 after H_2_O_2_ treatment, six were predicted to be putatively involved in MDR. These six MFS genes were AALTg5693 (quinidine resistance, log2FC 2.8), AALTg9681 (benomyl/methotrexate resistance, log2FC 3.4), AALTg6207 (multidrug resistance, log2FC 2.5), AALTg10470 (multidrug resistance, log2FC 2.0), AALTg9513 (benomyl/methotrexate resistance, log2FC 3.1) and AALTg8610 (gliotoxin efflux transporter, log2FC 1.8). One ABC transporter gene AALTg8644 (log2FC 1.2) was also up-regulated and it showed a high level similarity to the ABC multidrug transporter Mdr2 in *A*. *fumigatus*^57^. Interestingly, most of the down-regulated MFS genes are associated with sugar transport, indicating that the absorption of sugar nutrients may be retarded under oxidative stress.

### P450 and ergosterol biosynthesis

Cytochrome P450 (CYP) proteins are a type of monooxygenases which play essential roles in the biosynthesis of secondary metabolites and in the detoxification of toxic compounds^58^. We identified 13 CYPs whose expression was induced or repressed after H_2_O_2_ treatment (Supplementary Table S12). Specially, the 14-alpha sterol demethylase *ERG11B* gene (AALTg7699), which plays an essential role in ergosterol biosynthesis and drug resistance in many fungi^59^,^60^, showed a decreased expression by about 2.5 fold. Other genes required in ergosterol biosynthesis were then investigated. Interestingly, AALTg8874 (Sterol desaturase family, log2FC 1.5), AALTg10933 (sterol O-acyltransferase, log2FC 1.0), AALTg6701(Delta(7)-sterol 5(6)-desaturase, ERG3, log2FC -2.6) and AALTg1316 (squalene epoxidase, log2FC -2.0) showed changes in transcriptional level during H_2_O_2_ stress. Furthermore, the sterol regulatory element binding Sre1(AALTg8325, log2FC -1.1), which was reported to activate genes required for sterol biosynthesis under low oxygen, was significantly down-regulated^61^. In another experiment, a distinct expression pattern of sterol synthesis genes in response of *Cryptococcus neoformans* to H_2_O_2_ treatment was found, in which *ERG11*was induced while *ERG3* did not alter the transcript level at 30 min after H_2_O_2_ treatment^36^. These results may indicate a cross-talk between ergosterol biosynthesis and ROS resistance. However, the underlying mechanisms for the putative relationships between the two pathways may be different among fungi.

### HSPs and ubiquitin

Some general stress response proteins such as ubiquitin and heat shock proteins (HSPs) are known to play critical roles in fungal survival under various stresses. HSPs are constitutively expressed but can be induced to high levels under certain stresses in all living organisms. These proteins primarily function as molecular chaperones and maintain protein homeostasis in routine biological processes and under various stressful conditions^62^. For example, HSPs positively respond to ROS accumulation caused by thermal stress in yeast^63^. In this investigation, we found that 5 HSP40 (AALTg4905, log2FC 1.1, AALTg10836, log2FC 3.0, AALTg2843, log2FC 1.7, AALTg5344, log2FC 1.5 and AALTg5883, log2FC 1.1) and 2 HSP12 (AALTg4088, log2FC 4.2 and AALTg5164, log2FC 4.0) increased their level of mRNA expression in the H_2_O_2_ stress condition (Supplementary Table S13). Considering that the expression of HSPs may be specific to different conditions, these HSPs may be involved in H_2_O_2_ tolerance in *A*. *alternata*. Degradation of proteins by the ubiquitin dependent proteosome pathway is vital for maintaining cellular homeostasis. In mammalian cells, permanently oxidized proteins are recycled through the ubiquitin dependent proteasomal pathway^64^. Similar mechanism was also found in *C*. *neoformans* as the amount of ubiquitin conjugated proteins in the cell lysate is positively correlated the H_2_O_2_ concentration^36^. Besides, previous studies have found that both the *ubc8* deletion strain in *C*. *neoformans* and the *ubi4* deletion strain in *Candida albicans* displayed increased sensitivity to H_2_O_2_^65^,^66^. Through comparative transcriptome analysis, two ubiquitin-conjugating enzymes (AALTg6960, log2FC 1.8 and AALTg4835, log2FC 1.5) and one E3 ubiquitin-protein ligase (AALTg10966, log2FC 1.8) showed increased expression in response to H_2_O_2_ stress, suggesting a potential role of ubiquitin related processes in oxidative stress tolerance in *A*. *alternata*.

## Conclusions

In this study, we sequenced the genome of a tangerine pathotype of the plant fungal pathogen *A*. *alternata* Z7 and identified 19 novel genes associated with the biosynethesis of ACT. Ten genes were only found in the tangerine pathotype and they likely represented host-specificity determinants. Our analyses not only revealed the putative molecular basis of ACT biosynthesis but also provided potential molecular signatures for developing new methods of rapidly and efficiently detecting the tangerine pathotype of *A*. *alternata*. We also performed comparative global transcriptional studies of *A*. *alternata* Z7 to H_2_O_2_ stress and provided a broad-based analysis of gene expression linked to ROS resistance. The transcriptome data presented here will pave the way for future research on detoxification mechanisms of *A*. *alternata* towards ROS and further help reveal the underlying pathogenic mechanisms of this economically important fungal pathogen.

## Methods

### Genome sequencing and assembly

*A*. *alternata* strain Z7 was selected for genome sequencing using the long reads PacBio technology and the HiSeq 2000 platform^67^. A total of 1.6 Gb PacBio data, 1.1 Gb pair-end data and 4.0 Gb mate-pair data were generated in the sequencing process, which correspond to ~200 fold of sequence depth. The genome assembly was accomplished following a previously used method with the HGAP.2 assembler and the CLC Genomics Workbench program^68^,^69^. The assembled *A*. *alternata* Z7 genome has been deposited in GenBank under the accession number LPVP00000000 and genome information of other *Alternaria* species was downloaded from the *Alternaria* genomes database^70^.

### Gene prediction and annotation

*Ab initio* gene predictions of the genomic sequences of *Alternaria* species were performed with a combination of Augustus and GeneMark-ES^71^,^72^. The resulting prediction was refined using TopHat2 and Cufflinks on the RNA-seq libraries^73^,^74^. These predicted genes were primarily annotated based on BLASTp search against the NCBI (http://www.ncbi.nlm.nih.gov/) nr database from 13/06/15(*E* < 1 × 10^−5^ identity >25%, query coverage >50%). The tRNAs were identified by the tRNAscan-SE program^75^ and genome repetitive elements were defined using RepeatMasker^76^.

### Synteny, orthology and phylogenomic analysis

Syntenic analysis was performed by Circos with an identity and length cutoff set at 80% and 10 kb, respectively^77^. Orthologous gene relationship among species were determined using OrthMCL and reciprocal BLASTp with identity >50% and query coverage >50%^78^. The amino acid sequences from 200 random selected orthologous groups with only one gene in each species were concatenated and aligned with ClustalW2^79^. The maximum likelihood phylogenetic tree was subsequently built with the program MEGA6 using the Jones-Taylor-Thornton (JTT) model^80^. Statistical support for the phylogenetic tree was performed by non-parametric bootstrap analysis with 1000 replicates.

### Protein family classifications

The whole genome protein families were classified by InterproScan and Pfam analysis^81^,^82^. Fungal secondary metabolite pathways were predicted using the web-based analytical tool SMURF^83^.

### Transcriptome analysis

The *A*. *alternata* Z7strain was grown in liquid PDB at 25 °C in a shaker incubator for 2 days. H_2_O_2_ was added to the cultures to the final concentration of 15 mM with shaking for 30 min. The pure culture of *A*. *alternata* Z7 was used as a negative control. Mycelia were then collected for total RNA extraction using an AxyPrep^TM^ multisource total RNA miniprep kit. RNA-Seq was conducted for two biological replicates of each sample. The libraries were performed using an IlluminaTruSeq RNA Sample Preparation Kit and were sequenced on an Illumina Hiseq 2000 platform, generating 50 bp single-end reads. Index of the *A*. *alternata* Z7 genome was built using Bowtie2 and clean reads were mapped to the reference genome using TopHat2^74^,^84^. The reads numbers mapped to each gene was counted by HTSeq and the resulting transcript count tables were subjected to DESeq R package for differential expression analysis^85^,^86^. Transcripts with an adjusted P value less than 0.01 and a log2 (Fold change) greater than 1 were determined as differentially expressed. The differentially expressed genes were annotated by blast search against the NCBI nr databases. Gene Ontology (GO) enrichment analysis of DEGs was conducted using Blast2GO^87^. The transcriptome data reported in this study have been deposited in NCBI’s Sequence Read Archive (SRA) with accession number SRP071688.

### qRT-PCR analysis

To validate the transcriptome data obtained by RNA sequencing, qRT-PCR was carried out on eighteen *A*. *alternata* DEGs. 10 μg of each RNA sample was used for reverse transcription with the Prime Script RT reagent kit (TakaRa Biotechnology, Co., Dianlian, China). Relative expression of the selected genes was quantified in triplicate on a 7300 Real Time PCR system (ABI, USA). Primers used in this study were listed in Supplementary Table S14. The actin-encoding gene (KP341672) was used as an internal control and the resulting data was normalized using the comparative 2^−ΔΔCT^ as described previously^59^.

## Additional Information

**How to cite this article**: Wang, M. *et al*. Genomic and transcriptomic analyses of the tangerine pathotype of *Alternaria alternata* in response to oxidative stress. *Sci. Rep*. **6**, 32437; doi: 10.1038/srep32437 (2016).

## Supplementary Material

Supplementary Information

## Figures and Tables

**Table 1:** Assembly statistics for the *A*. *alternata* Z7 genome.

Features	*A*. *alternata* Z7
Genome size (Mb)	34.41
Number of contigs	161
N50 contig (kb)	1182
GC content (%)	51.0
Protein-coding genes	12062
Gene density (number of genes per Mb)	350
Mean gene length (bp)	1726
Mean number of exons per gene	2.8
Mean number of introns per gene	1.8
Percentage of genes without intron (%)	24.3
Repeat rate (%)	0.55
tRNA genes	115

**Table 2:** Up-regulation of genes encoding antioxidant compounds and enzymes after treatment with H_2_O_2_ (treated vs untreated).

locus_tag	log2FoldChange	FDR	Description
Peroxidase			
AALTg8994	4.33857956	2.20E-08	catalase
AALTg9933	4.322927007	8.58E-64	catalase
AALTg9032	3.359508277	2.39E-24	catalase
AALTg1523	3.760825042	1.69E-61	catalase-peroxidase
AALTg3786	2.822788592	1.60E-37	atypical 2-cysteine peroxiredoxin
AALTg10951	2.458733916	1.27E-23	1-cysteine peroxiredoxin
AALTg1812	2.266842651	5.55E-24	aypical 2-cysteine peroxiredoxin
AALTg7446	2.20816875	1.82E-22	atypical 2-cysteine peroxiredoxin
AALTg2814	2.441798208	1.27E-28	ascorbate peroxidase
AALTg11795	1.687903896	1.19E-10	carboxymuconolactone decarboxylase
AALTg3871	1.137861893	7.04E-06	hybridascorbate-cytochrome c peroxidase
Thioredoxin			
AALTg9686	2.60960513	1.49E-30	cop c 2-like protein
AALTg5799	1.032626507	2.40E-04	protein disulfide isomerase
AALTg5389	2.447386034	1.84E-18	trans-aconitate 2-methyltransferase
AALTg3416	2.571512121	6.63E-24	thioredoxin
AALTg2332	1.397281417	1.95E-06	capsule polysaccharide biosynthesis
AALTg11612	3.478028769	1.48E-33	thioredoxin
AALTg11329	1.567747404	1.17E-06	mitochondrialthioredoxin
Glutathione metabolism			
AALTg2809	4.33941433	1.12E-53	glutathione reductase, ec:1.8.1.7
AALTg5306	1.580570108	2.85E-07	glutathione S-transferase, ec:2.5.1.18
AALTg11609	2.304438673	1.77E-26	6-phosphogluconate dehydrogenase, ec:1.1.1.44
AALTg8364	1.67657098	1.27E-09	6-phosphogluconate dehydrogenase, ec:1.1.1.44
AALTg10627	1.262274201	9.85E-06	gamma-glutamyltranspeptidase 1 precursor, ec:2.3.2.2
AALTg6836	1.000063496	1.38E-05	glucose-6-phosphate 1-dehydrogenase,ec:1.1.1.49
AALTg1812	2.266842651	5.55E-24	Typical 2-cysteine peroxiredoxin, TryP, ec:1.11.1.15
AALTg10951	2.458733916	1.27E-23	1-cysteine peroxiredoxin, TryP, ec:1.11.1.15
AALTg41	2.314221216	1.74E-22	glutathione peroxidase, ec:1.11.1.9
AALTg6695	1.627714391	4.20E-08	glutathione synthetase large chain, ec:6.3.2.3
AALTg8327	1.366109598	3.19E-07	glutamate-cysteine ligase, ec:6.3.2.2
