# Supplementary material for: Genomic and transcriptomic analyses of the tangerine pathotype of Alternaria alternata in response to oxidative stress
Source: Sci Rep. 2016 Sep 1;6:32437. doi: 10.1038/srep32437 (PMC5007530; doi:10.1038/srep32437)
Supplement: Supplementary Information [file srep32437-s1.pdf]

Genomic and transcriptomic analyses of the tangerine pathotype of *Alternaria alternata* in response to oxidative stress

Mingshuang Wang[1], Xuepeng Sun[2], Dongliang Yu[3], Jianping Xu[4], Kuangren Chung[5]and Hongye Li[1]*

[1]Institute of Biotechnology, Zhejiang University, Hangzhou 310058, China;

[2]Boyce Thompson Institute, Cornell University, Ithaca14850, USA;

[3]College of Life and Environmental Sciences, Hangzhou Normal University, Hangzhou 310018, China;

[4]Department of Biology, McMaster University, Hamilton, Ontario L8S 4K1, Canada;

[5]Department of Plant Pathology, National Chung-Hsing University, Taichung 40227, Taiwan

***Corresponding authors:** Hongye Li

E-mail address: hyli@zju.edu.cn(H. Li)

Supplementary figures and tables

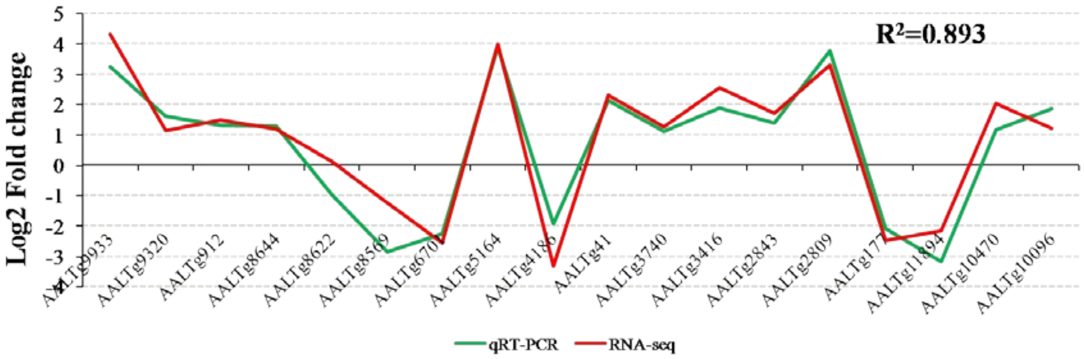

Supplementary Fig. S1 Validation of the RNA-seq data using qRT-PCR.

Supplementary Table S1 Genome structures of *Alternaria* species in terms of gene density.

Species	Coding genes	Genome size	Gene density
*Alternaria_alternata*_ATCC11680	11946	33752310	354
*Alternaria_alternata*_ATCC66891	11756	33236566	354
*Alternaria_alternata*_ATCC66982	11795	33465085	352
*Alternaria_alternata*_Z7	12049	34361435	351
*Alternaria_arborescens*_EGS128	11815	33889384	349
*Alternaria_brassicicola*_ATCC96836	10206	30291099	337
*Alternaria_capsici*_BMP0180	10974	33957558	323
*Alternaria_carthami*_BMP1963	11657	34455417	338
*Alternaria_citriarbusti*_BMP2343	12060	34124852	353
*Alternaria_crassa*_BMP0172	11128	35027983	318
*Alternaria_dauci*_BMP0167	11241	32148253	350
*Alternaria_destruens*_BMP0317	11331	41834136	271
*Alternaria_fragariae*_BMP3062	11733	33205225	353
*Alternaria_gaisen*_BMP2338	12774	34581883	369
*Alternaria_limoniasperae*_BMP2335	12300	35112645	350
*Alternaria_longipes*_BMP0313	12439	36313125	343
*Alternaria_macrospora*_BMP1949	11446	31742013	361
*Alternaria_mali*_BMP3063	12088	34085913	355
*Alternaria_mali*_BMP3064	12156	34664851	351
*Alternaria_porri*_BMP0178	11275	31231759	361
*Alternaria_solani*_BMP0185	11930	31649186	377
*Alternaria_tagetica*_BMP0179	11347	35064290	324
*Alternaria_tangelonis*_BMP2327	12017	33997552	353
*Alternaria_tenuissima*_BMP0304	11798	33516964	352
*Alternaria_tomatophila*_BMP2032	11893	34116351	349
*Alternaria_turkisafria*_BMP3436	12122	34016511	356

Supplementary Table S2 Genes unique to the tangerine pathotype of *Alternaria*.

Group id	*A.alternata* Z7	*A.citriarbusti*	*A.tangelonis*	*A.turkisafria*
prefix08213	AALTg11736	cit3|11952	tan7|11875	tur6|11833
prefix01447	AALTg11738	cit3|11921	tan7|11813	tur6|11763
prefix20298	AALTg11739	cit3|11920	tan7|11814	tur6|11764
prefix11992	AALTg11745	cit3|12030	tan7|11982	tur6|11517
prefix04636	AALTg11747	cit3|11859	tan7|11720	tur6|11519
prefix14311	AALTg11749	cit3|10964	tan7|10142	tur6|11365
prefix00448	AALTg11750	cit3|10965	tan7|10141	tur6|11220
prefix15510	AALTg11752	cit3|11831	tan7|11664	tur6|10299
prefix13275	AALTg11758	cit3|11314	tan7|11800	tur6|11842
prefix06193	AALTg12031	cit3|11992	tan7|11931	tur6|11867

Supplementary Table S3 Numbers of backbone genes involved in secondary metabolism in Z7 and selected fungi.

Species	PKS	NRPS	DMAT	Hybrid	PKS-like	NRPS-like	total
A.alternata Z7	12	7	0	0	1	5	25
G.graminicola	37	5	6	4	1	7	60
M.grisea	27	16	8	2	0	16	69
F.oxysporum	9	7	2	2	2	12	34
F.verticillioides	11	7	1	2	1	11	33
F.graminearum	14	10	0	1	1	11	37
N.haematococca	12	8	1	1	1	4	27
S.sclerotiorum	15	5	1	0	2	5	28

Supplementary Table S4 Secondary metabolites biosynthetic gene clusters in *A.alternata* Z7.

Cluster	Chromosome-Contig	backbone enzyes	gene	predicted products
Cluster:1	29	NRPS-Like	AALTg10556	unknown
Cluster:2	30	NRPS	AALTg10644	unknown
Cluster:3	48	NRPS	AALTg11749	ACT-toxin
Cluster:4	2	PKS-Like	AALTg1495	unknown
Cluster:5	2	PKS	AALTg1859	unknown
Cluster:6	3	PKS	AALTg2689	unknown
Cluster:7	3	PKS	AALTg2730	unknown
Cluster:8	4	PKS	AALTg2932	alternariol and alternariol-9-methyl ether
Cluster:9	1	PKS	AALTg409	alternapyrone
Cluster:10	6	NRPS	AALTg4616	unknown
Cluster:11	7	NRPS	AALTg5056	unknown
Cluster:12	10	PKS	AALTg6337	unknown
Cluster:13	10	NRPS	AALTg6627	unknown
Cluster:14	13	NRPS-Like	AALTg7486	unknown
Cluster:15	1	NRPS	AALTg869	dimethyl coprogen
Cluster:16	17	NRPS-Like	AALTg8711	unknown
Cluster:17	20	NRPS-Like	AALTg9348	unknown
Cluster:18	21	NRPS-Like	AALTg9403	unknown

Supplementary Table S5 The host specific ACT-toxin gene cluster in *A.alternata* Z7.

Number	Gene id*	ACT-toxin gene	Length(aa)	Gene Ontology:' molecular function	*A.citriarbusti*	*A.tangelonis*	*A.turkisafria*	Other pathotypes
a	AALTg12032	acyl-CoA dehydrogenase	349	acyl-CoA dehydrogenase activity; flavin adenine dinucleotide binding	+	+	+	+
**B**	AALTg12031	enoylreductase ACTTS2	343	zinc ion binding; oxidoreductase activity	+	+	+	-
**C**	AALTg11736	monooxygenase	608	NADP binding; flavin adenine dinucleotide binding; N,N-dimethylanilinemonooxygenase activity	+	+	+	-
d	AALTg11737	oxidoreductase	298	oxidoreductase activity	-	-	-	-
**E**	AALTg11738	hypothetical protein	436		+	+	+	-
**F**	AALTg11739	Thioesterase	216	hydrolase activity, acting on ester bonds	+	+	+	-
g	AALTg11740	acyl-CoA synthetase	578	catalytic activity	+	+	+	+
h	AALTg11741	hydroxymethylglutaryl-CoA synthase	404	hydroxymethylglutaryl-CoA synthase activity	+	+	+	+
i	AALTg11742	transposase	299	DNA binding	+	-	-	-
j	AALTg11743	hydrolase ACTT2	232		+	+	+	+
k	AALTg11744	hypothetical protein	139		-	-	-	-
**L**	AALTg11745	reverse transcriptase	208	nucleic acid binding	+	+	+	-
m	AALTg11746	ribonuclease H	610	nucleic acid binding; protein dimerization activity	+	+	+	+
**N**	AALTg11747	hypothetical protein	304		+	+	+	-

o	AALTg11748	metalloprotease	410		+	+	+	+
**P**	AALTg11749	non-ribosomal peptide synthetase	1404	catalyticactivity;phosphopantetheine binding	+	+	+	-
**Q**	AALTg11750	polyketide synthase ACTTS3	2457	catalytic activity; phosphopantetheine binding	+	+	+	-
r	AALTg11751	acyl-CoA synthetase ACTT5	475	catalytic activity	+	+	+	+
**S**	AALTg11752	hypothetical protein	297		+	+	+	-
t	AALTg11753	hypothetical protein	194		+	+	+	+
u	AALTg11754	Zn(II)$_2$Cys$_6$ transcription factor ACTTR	428	zinc ion binding; RNA polymerase II transcription factor activity, sequence-specific DNA binding	+	+	+	+
v	AALTg11755	HMG-CoA hydrolase ACTT3	296	catalytic activity	+	+	+	+
w	AALTg11756	Zn(II)$_2$Cys$_6$ transcription factor	545		+	+	+	+
x	AALTg11757	polyketide synthase	2349	catalytic activity; phosphopantetheine binding	+	+	+	+
**Y**	AALTg11758	cytochrome P450 monooxygenase	439	iron ion binding; oxidoreductase activity; heme binding	+	+	+	-

'*':The ACT-toxin gene cluster is located in two contigs, both contigs overlap 2734bp sequence at one end. '+'/'-': Orthologs present/absent in the corresponding fungus. Capital letters in column 'Number' represent genes uniquely present in the tangerine pathotype of *A.alternata*.

Supplementary Table S6 Distribution of CAZymes according to CAZy family among different fungi.

Species	A. fragariae	A. gaisen	A. limoniasperae	A. longipes	A.alternata_z7	A.arborescens	A.brassicicola	A.mali	B.uckeliana	F.graminearum	M.grisea	P.chrysogenum	P.tritici-repentis
AA1	11	9	8	10	10	11	7	9	0	14	15	2	11
AA11	0	0	0	0	0	0	0	0	2	4	7	9	0
AA12	0	0	0	0	0	0	0	0	0	2	4	0	0
AA13	0	0	0	0	0	0	0	0	1	1	1	2	0
AA2	2	2	1	1	2	2	2	1	0	3	5	0	2
AA3	30	24	28	30	27	34	11	30	5	20	21	1	21
AA4	4	4	5	5	5	5	2	5	1	1	0	1	4
AA5	2	1	2	1	2	1	0	1	3	5	4	2	2
AA6	0	0	0	0	0	0	0	0	1	1	1	1	0
AA7	17	17	14	16	17	17	10	16	1	4	2	0	15
AA8	0	0	0	0	0	0	0	0	0	3	7	0	0
AA9	19	16	24	21	23	23	15	21	9	13	24	4	17
CBM1	5	4	5	6	5	5	4	6	1	1	3	0	3
CBM13	4	2	2	1	3	3	2	1	0	1	0	0	2
CBM14	0	0	0	0	0	0	0	0	0	0	0	1	0
CBM18	0	0	0	0	0	1	1	0	0	3	15	2	0
CBM2	1	1	1	0	0	0	0	0	0	0	0	0	0
CBM20	1	1	1	1	1	2	1	1	0	0	0	0	2
CBM21	1	1	1	1	1	1	0	1	1	2	1	1	1
CBM48	0	0	0	0	0	0	0	0	2	2	2	1	1
CBM5	0	0	0	0	0	1	0	0	0	0	0	0	1
CBM50	2	1	2	3	1	2	0	3	1	9	7	5	0
CBM52	0	0	0	0	0	0	0	0	0	0	1	0	0
CBM63	0	0	0	0	0	0	0	0	1	3	1	1	0

CE1	2	0	1	1	1	2	2	1	2	4	9	1	1
CE10	28	23	30	31	28	25	22	31	0	0	0	0	22
CE12	1	1	1	1	1	1	1	1	3	3	3	2	0
CE14	0	0	0	0	0	0	0	0	0	0	0	0	1
CE15	0	0	0	0	0	0	0	0	0	0	1	1	0
CE16	0	0	0	0	0	0	0	0	6	5	2	1	0
CE2	0	0	0	0	0	0	0	0	0	1	1	0	0
CE3	0	0	0	0	0	0	0	0	1	5	6	4	0
CE4	8	7	9	9	8	8	4	9	5	7	10	5	7
CE5	11	10	12	12	12	12	7	12	11	13	17	4	11
CE8	3	2	3	3	3	3	3	3	5	3	1	2	2
CE9	0	0	0	0	0	0	0	0	2	1	1	1	0
GH1	2	3	2	2	2	3	2	2	3	3	2	3	2
GH10	6	5	6	6	6	5	4	6	2	5	6	3	4
GH105	3	3	3	3	3	3	3	3	1	3	4	2	2
GH109	0	1	0	0	0	0	0	0	0	0	0	0	0
GH11	5	3	3	3	4	4	3	3	3	3	5	1	3
GH114	0	0	0	0	0	0	0	0	1	2	1	2	0
GH115	0	0	0	0	0	0	0	0	1	2	2	0	0
GH12	4	3	3	3	4	4	0	3	5	4	3	3	1
GH125	2	2	2	2	2	2	1	2	3	3	6	1	3
GH127	0	0	0	0	0	0	0	0	1	1	0	0	0
GH128	5	4	4	5	4	3	4	5	4	4	4	1	3
GH13	4	4	4	4	4	4	4	4	11	8	10	16	4
GH131	0	0	0	0	0	0	0	0	1	1	5	0	0
GH132	0	0	0	0	0	0	0	0	2	2	2	2	0

GH134	0	0	0	0	0	0	0	0	0	0	0	3	0
GH15	2	2	2	2	2	2	2	2	5	3	2	3	3
GH16	6	7	6	6	6	6	3	6	23	24	17	16	7
GH17	1	1	1	0	1	1	2	0	6	6	7	5	1
GH18	9	8	9	9	9	7	4	9	10	17	16	8	7
GH2	2	2	3	3	2	3	1	3	2	10	8	6	3
GH20	1	1	1	1	1	1	1	1	1	3	3	2	1
GH24	0	0	0	0	0	0	0	0	0	0	1	0	0
GH25	0	0	0	0	0	0	0	0	0	0	0	1	0
GH26	1	1	1	1	1	1	1	1	2	0	0	1	0
GH27	4	2	5	5	5	3	2	5	4	2	4	1	4
GH28	7	7	8	8	8	8	5	8	19	7	4	5	4
GH29	0	0	0	0	0	0	1	0	0	1	4	0	0
GH3	14	12	12	12	11	12	7	12	16	21	18	17	8
GH30	0	0	1	0	1	1	0	0	0	0	1	1	1
GH31	2	2	2	2	2	2	3	2	4	8	6	11	3
GH32	2	2	2	2	2	2	1	2	1	5	5	7	1
GH33	0	0	1	1	1	0	0	1	0	1	0	2	0
GH35	4	2	4	4	4	4	3	4	4	3	0	2	3
GH36	2	2	1	1	1	1	2	1	2	3	1	2	0
GH37	1	1	1	1	1	2	2	1	1	2	2	1	2
GH38	1	1	1	1	1	1	1	1	1	1	2	1	1
GH39	0	0	0	0	0	0	0	0	1	2	2	2	0
GH42	0	0	0	0	0	0	0	0	0	0	0	1	0
GH43	12	11	14	14	14	14	10	14	5	18	18	14	11
GH45	2	2	2	2	2	2	0	2	2	1	1	0	2

GH47	9	8	10	9	9	9	7	9	8	10	9	6	16
GH5	7	6	7	7	7	6	5	7	16	14	13	13	3
GH51	2	2	2	2	2	2	2	2	4	2	3	3	2
GH53	1	1	1	1	1	1	1	1	2	1	1	1	0
GH54	1	0	1	1	1	1	1	1	1	1	1	1	1
GH55	2	3	3	3	3	3	2	3	4	3	7	3	1
GH6	3	2	3	3	3	3	2	3	1	1	3	1	3
GH62	2	1	2	2	2	2	1	2	1	2	4	1	1
GH63	2	1	2	2	2	2	2	2	1	1	2	1	2
GH64	0	0	0	0	0	0	0	0	2	2	2	2	0
GH65	0	1	0	0	0	0	0	0	2	0	0	1	0
GH67	1	1	1	1	1	1	0	1	0	1	1	1	1
GH7	4	3	4	4	4	4	4	4	2	2	6	2	3
GH71	0	0	0	0	0	0	0	0	11	0	1	6	0
GH72	5	5	6	5	6	5	4	5	6	3	5	6	5
GH74	0	0	0	0	0	0	0	0	0	1	1	0	0
GH75	0	0	0	0	0	0	0	0	0	1	1	1	0
GH76	5	7	6	6	6	6	6	6	11	8	8	8	4
GH78	4	4	4	4	4	4	0	4	8	7	3	5	3
GH79	0	0	0	0	0	0	0	0	2	1	2	2	0
GH81	0	1	1	1	1	1	0	1	1	1	2	1	1
GH84	0	0	0	0	0	0	0	0	0	0	0	1	0
GH85	0	0	0	0	0	0	1	0	0	0	0	0	1
GH88	1	1	0	1	0	0	0	1	1	1	1	0	0
GH92	5	5	4	5	4	5	3	5	5	0	6	4	5
GH93	0	0	0	0	0	0	0	0	1	2	1	2	0

GH94	1	1	1	1	1	1	1	1	0	0	1	0	1
GH95	0	0	0	0	0	0	0	0	2	2	1	1	0
GT1	6	4	7	7	7	6	2	7	13	16	13	6	3
GT15	3	3	3	3	3	3	1	3	4	4	4	3	4
GT17	0	0	0	0	0	0	0	0	2	2	1	0	0
GT2	3	3	3	3	3	3	2	3	13	20	11	13	4
GT20	3	2	3	3	3	3	3	3	3	3	3	9	3
GT21	0	0	0	0	0	0	0	0	1	1	1	1	0
GT22	3	2	2	2	2	3	2	2	4	4	4	4	1
GT24	1	1	1	1	1	1	1	1	1	1	1	1	1
GT25	0	0	0	0	0	0	0	0	2	0	6	3	0
GT3	1	0	1	1	1	1	0	1	1	1	1	1	1
GT31	0	0	0	0	0	0	0	0	6	5	4	6	0
GT32	2	3	3	3	3	2	2	3	6	5	10	9	2
GT33	0	0	0	0	0	0	0	0	0	1	1	1	0
GT34	9	6	8	9	9	11	5	9	1	2	2	3	3
GT35	1	1	1	1	1	1	1	1	1	1	1	1	1
GT39	3	3	3	3	3	3	2	3	3	3	3	3	3
GT4	3	2	3	3	3	3	2	3	5	5	4	5	4
GT41	1	1	1	1	1	1	0	1	1	1	1	1	0
GT48	1	1	1	1	1	1	0	1	2	1	1	1	1
GT50	0	0	0	0	0	0	0	0	1	1	1	1	0
GT55	0	0	0	0	0	0	0	0	0	0	1	0	1
GT57	0	0	0	0	0	0	0	0	2	2	2	2	0
GT58	1	1	1	1	1	1	1	1	1	1	1	1	1
GT59	1	1	1	1	1	1	1	1	0	1	1	1	0

GT61	0	0	0	0	0	0	0	0	0	0	3	0	0
GT62	2	2	1	2	2	2	3	2	3	3	3	3	3
GT64	0	0	0	0	0	0	0	0	0	2	0	0	0
GT66	0	0	0	0	0	0	0	0	1	1	1	1	1
GT69	2	3	2	3	2	2	0	3	3	2	4	2	3
GT71	1	1	1	1	1	1	1	1	5	4	4	3	1
GT76	1	1	1	1	1	1	0	1	2	1	1	1	1
GT8	2	3	3	2	2	3	2	2	7	6	2	5	3
GT90	0	0	0	0	0	0	0	0	3	6	6	6	1
PL1	6	6	7	7	6	6	4	7	6	9	2	5	6
PL20	0	0	0	0	0	0	0	0	0	1	1	0	0
PL3	5	5	5	6	5	6	8	6	2	7	1	1	3
PL4	0	0	0	0	0	0	0	0	0	3	1	3	0
PL7	0	0	0	0	0	0	0	0	1	0	0	0	0
PL9	0	0	0	0	0	0	0	0	0	1	0	0	0

Supplementary Table S7 Differentially expressed genes that enriched in the category 'oxidoreductase activity'.

ID	log2FC	P-adj	annotation
AALTg3456	5.12	8.06E-43	2,4-dichlorophenol 6-monooxygenase
AALTg3691	4.73	2.05E-53	alcohol dehydrogenase
AALTg2809	4.34	1.12E-53	glutathione reductase
AALTg8994	4.34	2.20E-08	peroxisomal catalase
AALTg9933	4.32	8.58E-64	catalase A
AALTg5523	4.32	1.36E-40	alcohol dehydrogenase
AALTg11123	4.09	5.22E-18	Alcohol dehydrogenase superfamily zinc-containing
AALTg7769	4.02	3.92E-42	thioredoxin reductase
AALTg353	3.98	1.23E-42	3-chlorobenzoate-3,4-dioxygenase reductase subunit
AALTg215	3.83	8.86E-39	Aldo keto reductase
AALTg1523	3.76	1.69E-61	catalase-peroxidase
AALTg1561	3.68	5.75E-42	FAD-binding domain
AALTg398	3.67	4.10E-54	zinc-binding oxidoreductase
AALTg6718	3.64	5.82E-41	NAD dependent epimerase dehydratase family
AALTg2751	3.42	1.32E-05	nadph2:quinone reductase
AALTg9032	3.36	2.39E-24	mycelial catalase Cat1
AALTg1664	3.33	1.70E-03	catalase domain containing
AALTg275	3.32	2.94E-14	oxidoreductase
AALTg473	3.27	7.08E-04	cytochrome P450
AALTg9998	3.23	2.83E-06	hypothetical protein W97_01942
AALTg6800	3.19	8.97E-07	zinc alcohol dehydrogenase,
AALTg11797	3.17	3.79E-35	glucose oxidase
AALTg5137	3.16	1.84E-14	cytochrome P450 4A10
AALTg4968	3.15	1.57E-20	Dehydroquinate synthase
AALTg6835	2.97	7.01E-37	D-3-phosphoglycerate dehydrogenase
AALTg10949	2.95	1.07E-33	pyridine nucleotide-disulfide oxidoreductase class-2
AALTg4285	2.92	1.29E-20	FAD dependent oxidoreductase
AALTg4679	2.83	2.69E-06	quinone oxidoreductase
AALTg5416	2.83	2.43E-21	NADP-dependent leukotriene B4 12-hydroxydehydrogenase
AALTg11389	2.76	4.57E-34	FMN-binding split barrel-related
AALTg4863	2.76	4.70E-24	NADPH2 dehydrogenase
AALTg5757	2.76	1.49E-30	S-(hydroxymethyl)glutathione dehydrogenase
AALTg11995	2.66	1.57E-24	NADH-dependent flavin oxidoreductase
AALTg4107	2.61	7.04E-06	fad linked oxidase
AALTg319	2.59	4.06E-20	molybdopterin binding oxidoreductase
AALTg5326	2.47	1.84E-17	zinc-binding alcohol dehydrogenase
AALTg10951	2.46	1.27E-23	mitochondrial peroxiredoxin Prx1
AALTg2814	2.44	1.27E-28	cytochrome c peroxidase
AALTg5739	2.41	1.06E-22	tri-functional histidine biosynthesis

AALTg3043	2.40	7.87E-09	FAD binding domain
AALTg10228	2.39	1.22E-23	Peptide methionine sulfoxide reductase
AALTg41	2.31	1.74E-22	glutathione peroxidase 1
AALTg11609	2.30	1.77E-26	6-phosphogluconate dehydrogenase
AALTg1812	2.27	5.55E-24	peroxiredoxin TSA1
AALTg666	2.22	3.79E-10	FMN-dependent dehydrogenase
AALTg3134	2.19	5.98E-08	pisatin demethylase
AALTg7589	2.17	1.22E-13	Lathosterol oxidase
AALTg10668	2.17	7.05E-13	glutaryl- dehydrogenase
AALTg3306	2.14	1.80E-08	maleylacetate reductase
AALTg11345	2.13	8.47E-17	NADH-ubiquinone oxidoreductase 64 kDa subunit
AALTg4224	2.10	2.66E-06	precorrin 3B synthase
AALTg4587	2.10	4.71E-18	FMN-linked oxidoreductase
AALTg8230	2.09	4.02E-19	glycerol-3-phosphate dehydrogenase, mitochondrial precursor
AALTg3590	2.04	2.34E-08	alcohol dehydrogenase
AALTg7455	2.02	6.23E-13	L-lactate dehydrogenase (cytochrome)
AALTg8957	1.97	3.04E-04	alpha-ketoglutarate-dependent taurine dioxygenase
AALTg10478	1.95	5.75E-09	hypothetical protein SETTUDRAFT_134436
AALTg5382	1.91	6.85E-03	DNA repair family
AALTg10141	1.89	2.21E-09	L-lactate dehydrogenase
AALTg9951	1.87	1.76E-06	FAD dependent oxidoreductase
AALTg5763	1.82	3.09E-16	nadh-cytochrome b5 reductase
AALTg2786	1.81	3.47E-12	3-oxo-5-alpha-steroid 4-dehydrogenase
AALTg1647	1.81	7.56E-13	kynurenine 3-monooxygenase
AALTg9558	1.75	1.42E-03	FAD NAD(P)-binding domain-containing
AALTg409	1.73	4.62E-05	polyketide synthase
AALTg8354	1.73	7.56E-07	NAD-dependent malic enzyme 1
AALTg3682	1.73	5.34E-13	nadph-dependent medium chain alcohol dehydrogenase
AALTg6641	1.72	3.34E-06	L-galactose dehydrogenase (L-)
AALTg9044	1.72	1.43E-09	mitochondrial FAD-linked sulfhydryl oxidase ERV1
AALTg3897	1.69	9.48E-03	alcohol dehydrogenase
AALTg11795	1.69	1.19E-10	Carboxymuconolactone decarboxylase
AALTg8364	1.68	1.27E-09	6-phosphogluconate dehydrogenase C-terminal domain
AALTg3633	1.65	7.86E-03	ferric-chelate reductase
AALTg1841	1.64	4.76E-08	phosphoadenosine phosphosulfate reductase
AALTg11642	1.64	4.68E-11	alcohol dehydrogenase
AALTg5665	1.59	3.42E-03	oxidoreductase domain containing
AALTg5306	1.58	2.85E-07	glutathione transferase omega-1
AALTg2893	1.58	3.57E-08	FMN-dependent alpha-hydroxy acid dehydrogenase
AALTg1718	1.56	5.56E-11	aspartate-semialdehyde dehydrogenase
AALTg4194	1.54	9.38E-07	NADPH:adrenodoxin oxidoreductase, mitochondrial precursor
AALTg1566	1.53	2.71E-06	2-polyprenyl-6-methoxyphenol hydroxylase-like

			oxidoreductase
AALTg9783	1.53	1.36E-06	quinone oxidoreductase
AALTg8874	1.52	1.75E-04	sterol desaturase family
AALTg10309	1.44	3.12E-05	aflatoxin B1-aldehyde reductase
AALTg1441	1.42	1.52E-08	zinc-binding dehydrogenase
AALTg1795	1.40	3.27E-05	phytoene dehydrogenase
AALTg11153	1.37	1.50E-04	homogentisate 1,2-dioxygenase
AALTg4006	1.37	9.13E-08	Shikimate kinase
AALTg11209	1.37	3.19E-03	L-lactate dehydrogenase
AALTg8452	1.35	8.41E-05	ATP binding
AALTg2529	1.35	6.98E-05	NAD-specific glutamate dehydrogenase
AALTg717	1.29	7.04E-06	methionine-R-sulfoxide reductase
AALTg348	1.27	4.13E-05	sulfite reductase flavo component
AALTg3600	1.24	7.36E-04	amine oxidase
AALTg2498	1.23	5.23E-05	FAD dependent oxidoreductase
AALTg8160	1.21	7.97E-03	benzoate 4-monooxygenase cytochrome P450
AALTg1122	1.16	1.04E-03	pyridine nucleotide-disulphide oxidoreductase,
AALTg6527	1.14	5.16E-06	glycerol dehydrogenase
AALTg3871	1.14	7.04E-06	conserved hypothetical protein
AALTg2795	1.11	1.15E-04	aldo keto reductase
AALTg366	1.10	2.46E-03	cytochrome P450
AALTg2177	1.08	8.55E-03	flavin dependent monooxygenase
AALTg1283	1.07	4.07E-04	Succinic semialdehyde dehydrogenase
AALTg1758	1.05	7.48E-03	siroheme synthase
AALTg6836	1.00	1.38E-05	glucose-6-phosphate 1-dehydrogenase
AALTg4772	-1.02	1.85E-03	2-nitropropane dioxygenase
AALTg4384	-1.04	7.67E-05	formate dehydrogenase
AALTg198	-1.05	1.93E-04	FMN-linked oxidoreductase
AALTg7620	-1.07	1.50E-03	Cytochrome P450
AALTg4399	-1.11	9.00E-06	coproporphyrinogen III oxidase
AALTg8358	-1.16	2.41E-03	benzoate 4-monooxygenase cytochrome P450
AALTg7370	-1.17	2.36E-06	delta-1-pyrroline-5-carboxylate dehydrogenase, mitochondrial
AALTg7790	-1.19	9.61E-04	fumarate reductase
AALTg6572	-1.19	2.46E-05	trimethyllysine dioxygenase
AALTg8569	-1.24	1.34E-03	isovaleryl- dehydrogenase
AALTg7358	-1.30	1.80E-06	Cytochrome P450
AALTg7699	-1.31	1.45E-07	14-alpha sterol demethylase Cyp51B
AALTg3330	-1.32	5.41E-03	benzoate 4-monooxygenase cytochrome P450,
AALTg5369	-1.32	2.36E-04	NADP-dependent leukotriene B4 12-hydroxydehydrogenase
AALTg3232	-1.35	1.68E-04	D-amino-acid oxidase
AALTg8807	-1.42	1.52E-03	aldehyde dehydrogenase
AALTg7831	-1.42	3.74E-04	monooxygenase FAD-binding

AALTg6028	-1.43	4.94E-05	NADPH oxidase B
AALTg5948	-1.43	2.21E-05	acyl- dehydrogenase family
AALTg7698	-1.44	1.39E-03	oxidoreductase, 2-nitropropane dioxygenase family,
AALTg9303	-1.45	4.47E-03	sorbitol dehydrogenase
AALTg7278	-1.49	1.21E-07	flavin-binding monooxygenase
AALTg387	-1.51	9.44E-03	WSC domain-containing
AALTg8571	-1.54	2.97E-05	catechol dioxygenase
AALTg2155	-1.54	6.04E-05	2-nitropropane dioxygenase
AALTg7817	-1.67	2.11E-08	Fe II 2-oxoglutarate-dependent dioxygenase
AALTg9357	-1.75	7.79E-04	phenylacetate hydroxylase
AALTg4185	-1.79	8.75E-06	nitrate reductase [NADPH]
AALTg7195	-1.85	1.29E-04	FAD NAD-P-binding domain-containing
AALTg3225	-1.85	5.83E-08	isoamyl alcohol oxidase
AALTg10600	-1.89	2.30E-06	FAD-linked sulfhydryl oxidase ERV2
AALTg9891	-1.90	5.46E-09	dimethylaniline monooxygenase 2
AALTg9212	-1.91	6.94E-05	alcohol dehydrogenase
AALTg5338	-1.97	8.95E-14	fad binding domain-containing
AALTg1316	-2.01	2.96E-15	squalene epoxidase
AALTg9280	-2.01	3.85E-03	sarcosine oxidase
AALTg10932	-2.05	1.30E-03	3-hydroxyacyl- dehydrogenase
AALTg10173	-2.07	8.56E-04	GMC oxidoreductase
AALTg11894	-2.09	5.54E-05	bifunctional P-450:NADPH-P450 reductase
AALTg5241	-2.39	1.00E-27	FAD dependent oxidoreductase
AALTg6244	-2.54	2.03E-21	acyl- desaturase
AALTg6701	-2.56	4.80E-24	C-5 sterol desaturase
AALTg4186	-3.32	6.76E-25	nitrite reductase
AALTg5212	-3.53	6.22E-44	uricase

Supplementary Table S8 Differentially expressed genes encoding kinases.

ID	Subfamily	log2FC	padj	annotation
AALTg3688	CMGC_group2_multiple	2.10	6.29E-09	kinase domain-containing
AALTg8919	CAMK_group6_multiple	1.98	3.78E-15	nitrogen permease reactivator
AALTg8419	CAMK_group5_multiple	1.92	3.72E-13	Ca2+ calmodulin-dependent kinase
AALTg3740	CAMK_group7_multiple	1.27	4.26E-06	serine threonine kinase SNF1p
AALTg7163	AGC_group2_multiple	1.23	4.23E-06	serine threonine- kinase nrc-2
AALTg10096	CMGC_group1_multiple	1.21	2.14E-06	map kinase Hog1
AALTg6950		1.18	7.76E-04	serine threonine kinase
AALTg3039	STE_group3_multiple	1.09	2.53E-04	STE STE7 MKK kinase
AALTg6372	CMGC_group4_multiple	1.06	2.63E-05	serine kinase Sky1
AALTg8652	AGC_group4_multiple	1.03	7.03E-04	histidine kinase HHK1p
AALTg6232	AGC_group4_multiple	-2.25	3.94E-12	serine threonine- kinase 12
AALTg3632	STE_group3_multiple	-1.86	1.26E-04	calcium calmodulin-dependent kinase type 2 beta chain
AALTg9127	CMGC_group2_multiple	-1.80	3.11E-04	serine threonine kinase Hsk1
AALTg2090	AGC_group2_multiple	-1.73	9.43E-04	serine threonine- kinase cbk1
AALTg9028	AGC_group5_multiple	-1.16	1.27E-03	checkpoint kinase
AALTg2778	AGC_group5_multiple	-1.06	6.13E-03	IKS kinase

Supplementary Table S9 Transcription factors in the genome of *A.alternata*.

Gene ID	Subfamily	Gene ID	Subfamily	Gene ID	Subfamily	Gene ID	Subfamily
AALTg640	ARID	AALTg5836	HTH_psq	AALTg6166	Zn_clus	AALTg193	Zn_clus
AALTg5157	ARID	AALTg11698	HTH_psq	AALTg5549	Zn_clus	AALTg3026	Zn_clus
AALTg2163	ARID	AALTg10904	KilA-N	AALTg962	Zn_clus	AALTg10925	Zn_clus
AALTg497	AT_hook	AALTg34	KilA-N	AALTg8907	Zn_clus	AALTg3444	Zn_clus
AALTg912	bZIP_1	AALTg11863	Myb_DNA-binding	AALTg7025	Zn_clus	AALTg968	Zn_clus
AALTg6125	bZIP_1	AALTg4660	Myb_DNA-binding	AALTg9417	Zn_clus	AALTg5383	Zn_clus
AALTg3227	bZIP_1	AALTg7341	Myb_DNA-binding	AALTg4691	Zn_clus	AALTg10980	Zn_clus
AALTg9614	bZIP_1	AALTg4949	Myb_DNA-binding	AALTg8967	Zn_clus	AALTg8016	Zn_clus
AALTg7253	bZIP_1	AALTg1813	Myb_DNA-binding	AALTg8890	Zn_clus	AALTg7121	Zn_clus
AALTg3816	bZIP_1	AALTg12028	Myb_DNA-binding	AALTg9074	Zn_clus	AALTg3883	Zn_clus
AALTg5243	bZIP_1	AALTg135	Myb_DNA-binding	AALTg9150	Zn_clus	AALTg10290	Zn_clus
AALTg6239	bZIP_1	AALTg8710	Myb_DNA-binding	AALTg8164	Zn_clus	AALTg9979	Zn_clus
AALTg10928	bZIP_1	AALTg4507	Myb_DNA-binding	AALTg597	Zn_clus	AALTg11686	Zn_clus
AALTg1854	bZIP_1	AALTg134	Myb_DNA-binding	AALTg11415	Zn_clus	AALTg2676	Zn_clus
AALTg7239	bZIP_1	AALTg4214	Myb_DNA-binding	AALTg3007	Zn_clus	AALTg2093	Zn_clus
AALTg5000	bZIP_1	AALTg4207	Myb_DNA-binding	AALTg12014	Zn_clus	AALTg483	Zn_clus
AALTg11016	bZIP_1	AALTg6153	NDT80_PhoG	AALTg331	Zn_clus	AALTg2353	Zn_clus
AALTg5461	bZIP_1	AALTg4760	NDT80_PhoG	AALTg2025	Zn_clus	AALTg5912	Zn_clus
AALTg9986	bZIP_1	AALTg10851	RFX_DNA_binding	AALTg8206	Zn_clus	AALTg6005	Zn_clus
AALTg12013	CENP-B_N	AALTg5670	SRF-TF	AALTg6118	Zn_clus	AALTg10943	Zn_clus
AALTg3278	CENP-B_N	AALTg3655	SRF-TF	AALTg3400	Zn_clus	AALTg1223	Zn_clus
AALTg6265	CP2	AALTg11081	zf-C2H2	AALTg9105	Zn_clus	AALTg2303	Zn_clus
AALTg10545	Fork_head	AALTg925	zf-C2H2	AALTg3145	Zn_clus	AALTg7896	Zn_clus
AALTg8403	Fork_head	AALTg2998	zf-C2H2	AALTg1363	Zn_clus	AALTg8898	Zn_clus

AALTg3380	Fork_head	AALTg8855	zf-C2H2	AALTg5016	Zn_clus	AALTg1404	Zn_clus
AALTg3730	Fork_head	AALTg3586	zf-C2H2	AALTg11091	Zn_clus	AALTg8266	Zn_clus
AALTg4320	GATA	AALTg8208	zf-C2H2	AALTg11405	Zn_clus	AALTg6414	Zn_clus
AALTg5596	GATA	AALTg11864	zf-C2H2	AALTg7922	Zn_clus	AALTg11974	Zn_clus
AALTg8326	GATA	AALTg9410	zf-C2H2	AALTg3166	Zn_clus	AALTg4807	Zn_clus
AALTg9258	GATA	AALTg11124	zf-C2H2	AALTg5733	Zn_clus	AALTg8375	Zn_clus
AALTg5872	GATA	AALTg8118	zf-C2H2	AALTg10394	Zn_clus	AALTg8483	Zn_clus
AALTg11365	GATA	AALTg1605	zf-C2H2	AALTg10972	Zn_clus	AALTg11263	Zn_clus
AALTg9677	GATA	AALTg4467	zf-C2H2	AALTg6529	Zn_clus	AALTg8538	Zn_clus
AALTg8249	HLH	AALTg8698	zf-C2H2	AALTg4795	Zn_clus	AALTg8172	Zn_clus
AALTg7890	HLH	AALTg6851	zf-C2H2	AALTg127	Zn_clus	AALTg4441	Zn_clus
AALTg5726	HLH	AALTg4823	zf-C2H2	AALTg10806	Zn_clus	AALTg10688	Zn_clus
AALTg11151	HLH	AALTg5513	zf-C2H2	AALTg7641	Zn_clus	AALTg5939	Zn_clus
AALTg7108	HLH	AALTg9430	zf-C2H2	AALTg9816	Zn_clus	AALTg9541	Zn_clus
AALTg9883	HLH	AALTg10348	zf-C2H2	AALTg7249	Zn_clus	AALTg11283	Zn_clus
AALTg4560	HLH	AALTg4312	zf-C2H2	AALTg4438	Zn_clus	AALTg9129	Zn_clus
AALTg9274	HLH	AALTg6755	zf-C2H2	AALTg7586	Zn_clus	AALTg8822	Zn_clus
AALTg4433	HLH	AALTg9740	zf-C2H2	AALTg7282	Zn_clus	AALTg8613	Zn_clus
AALTg9188	HLH	AALTg8241	zf-C2H2	AALTg9163	Zn_clus	AALTg11861	Zn_clus
AALTg8325	HLH	AALTg5113	zf-C2H2	AALTg10558	Zn_clus	AALTg8635	Zn_clus
AALTg1832	HLH	AALTg1011	zf-C2H2	AALTg8265	Zn_clus	AALTg10251	Zn_clus
AALTg3713	HMG_box	AALTg2857	zf-C2H2	AALTg2310	Zn_clus	AALTg8267	Zn_clus
AALTg10579	HMG_box	AALTg10284	zf-C2H2	AALTg1190	Zn_clus	AALTg7084	Zn_clus
AALTg9435	HMG_box	AALTg1296	zf-C2H2	AALTg9284	Zn_clus	AALTg2685	Zn_clus
AALTg5202	HMG_box	AALTg10527	zf-C2H2	AALTg1721	Zn_clus	AALTg9841	Zn_clus
AALTg9267	HMG_box	AALTg10200	zf-C2H2	AALTg7682	Zn_clus	AALTg1569	Zn_clus

AALTg11859	HMG_box	AALTg65	zf-C2H2	AALTg7476	Zn_clus	AALTg9629	Zn_clus
AALTg11926	HMG_box	AALTg11938	zf-C2H2	AALTg6057	Zn_clus	AALTg1405	Zn_clus
AALTg5429	HMG_box	AALTg7015	zf-C2H2	AALTg11931	Zn_clus	AALTg4236	Zn_clus
AALTg2977	Homeobox	AALTg3874	zf-C2H2	AALTg11754	Zn_clus	AALTg7148	Zn_clus
AALTg10699	Homeobox	AALTg561	zf-C2H2	AALTg2810	Zn_clus	AALTg1514	Zn_clus
AALTg9443	Homeobox	AALTg9029	zf-C2H2	AALTg8539	Zn_clus	AALTg1947	Zn_clus
AALTg6581	Homeobox	AALTg4096	zf-C2H2	AALTg8816	Zn_clus	AALTg11210	Zn_clus
AALTg24	Homeobox	AALTg6703	zf-C2H2	AALTg3664	Zn_clus	AALTg8380	Zn_clus
AALTg785	Homeobox	AALTg6557	zf-C2H2	AALTg1072	Zn_clus	AALTg9167	Zn_clus
AALTg3476	Homeobox	AALTg4	zf-C2H2	AALTg7392	Zn_clus	AALTg9821	Zn_clus
AALTg6758	Homeobox	AALTg11727	zf-C2H2	AALTg8318	Zn_clus	AALTg8290	Zn_clus
AALTg8622	HSF_DNA-bind	AALTg2519	zf-C2H2	AALTg7054	Zn_clus	AALTg10177	Zn_clus
AALTg6971	HSF_DNA-bind	AALTg7495	zf-C2H2	AALTg177	Zn_clus	AALTg3102	Zn_clus
AALTg4838	HSF_DNA-bind	AALTg1236	zf-C2H2	AALTg2847	Zn_clus	AALTg9458	Zn_clus
AALTg3490	HTH_psq	AALTg3995	zf-C2H2	AALTg6558	Zn_clus	AALTg2686	Zn_clus
AALTg3772	HTH_psq	AALTg11041	zf-C2H2	AALTg5614	Zn_clus	AALTg7336	Zn_clus
AALTg2441	HTH_psq	AALTg3702	zf-C2H2	AALTg5909	Zn_clus	AALTg4183	Zn_clus
AALTg11742	HTH_psq	AALTg9572	zf-C2H2	AALTg6334	Zn_clus	AALTg303	Zn_clus
AALTg11054	HTH_psq	AALTg5661	Zn_clus	AALTg8531	Zn_clus	AALTg9231	Zn_clus
AALTg12029	HTH_psq	AALTg253	Zn_clus	AALTg10254	Zn_clus	AALTg57	Zn_clus
AALTg12006	HTH_psq	AALTg1552	Zn_clus	AALTg10137	Zn_clus		

Supplementary Table S10 Differentially expressed genes encoding transcription factors.

ID	Subfamily	log2FC	padj	annotation
AALTg3444	Zn_clus	2.77	5.51E-03	transcription factor Cys6
AALTg7922	Zn_clus	2.63	5.40E-04	fungal specific transcription factor domain-containing
AALTg4441	Zn_clus	2.36	3.65E-14	zinc finger transcription factor 1
AALTg8375	Zn_clus	2.04	1.53E-11	C6 zinc finger domain
AALTg2685	Zn_clus	2.04	2.74E-03	fungal specific transcription factor domain-containing
AALTg8380	Zn_clus	1.87	1.11E-10	c6 transcription
AALTg1813	Myb_DNA-binding	1.63	6.83E-05	MYB DNA-binding domain containing
AALTg6239	bZIP_1	1.63	1.77E-09	bzip transcription factor
AALTg3713	HMG_box	1.56	1.14E-05	hmg box
AALTg7476	Zn_clus	1.55	5.43E-03	C6 transcription factor
AALTg912	bZIP_1	1.49	6.12E-09	AP1-like transcription factor
AALTg11151	HLH	1.40	6.41E-05	HLH transcription factor (gamma)
AALTg8635	Zn_clus	1.34	5.33E-03	related to nitrate assimilation regulatory nirA
AALTg5383	Zn_clus	1.28	6.78E-06	Transcription factor fungi
AALTg8016	Zn_clus	1.15	4.65E-04	fungal specific transcription factor domain containing
AALTg9105	Zn_clus	1.09	1.07E-03	C6 finger domain
AALTg785	Homeobox	1.09	3.62E-05	homeobox and c2h2 transcription factor
AALTg8898	Zn_clus	1.08	4.33E-04	pathway-specific nitrogen regulator
AALTg8241	zf-C2H2	1.03	2.49E-03	Zinc finger C2H2-type
AALTg5113	zf-C2H2	-1.03	9.80E-04	DNA-binding creA
AALTg8325	HLH	-1.10	3.16E-04	sterol regulatory element binding Sre1
AALTg127	Zn_clus	-1.28	5.22E-03	transcription factor cys6
AALTg6755	zf-C2H2	-1.32	6.61E-05	transcription factor steA
AALTg2857	zf-C2H2	-1.52	4.49E-05	c2h2 type zinc finger domain-containing

AALTg9883	HLH	-1.68	4.03E-13	transcription factor bhlh
AALTg8326	GATA	-1.74	4.15E-05	GATA-type sexual development transcription factor
AALTg4507	Myb_DNA-binding	-1.82	5.10E-04	trichome differentiation GL1
AALTg10972	Zn_clus	-1.84	3.36E-07	hypothetical protein PTT_10983
AALTg9231	Zn_clus	-1.99	2.12E-05	hypothetical protein COCMIDRAFT_22048
AALTg11054	HTH_psq	-2.07	3.00E-03	transposase, partial
AALTg4207	Myb_DNA-binding	-2.15	1.25E-05	Myb-like DNA-binding domain
AALTg9274	HLH	-2.45	3.85E-29	hypothetical protein COCSADRAFT_90297
AALTg177	Zn_clus	-2.47	5.31E-14	c6 transcription factor

Supplementary Table S11 Differentially expressed genes encoding MFS and ABC transporters.

ID		log2FC	padj	annotation
AALTg1366	2.A.1	4.90	4.68E-52	MSF membrane transporter
AALTg8641	2.A.1	3.41	1.55E-11	allantoin permease
AALTg9681	2.A.1	3.36	1.15E-37	benomyl methotrexate resistance
AALTg3878	2.A.1	3.36	2.66E-31	general substrate transporter
AALTg6186	2.A.1	3.34	7.56E-05	citrinin biosynthesis transporter
AALTg1881	2.A.1	3.27	2.28E-20	MFS general substrate transporter
AALTg9513	2.A.1	3.11	8.55E-11	multidrug resistance
AALTg5693	2.A.1	2.83	2.33E-08	Quinidine resistance 2
AALTg3560	2.A.1	2.71	3.86E-27	major facilitator superfamily transporter
AALTg6492	2.A.1	2.59	4.81E-04	Major facilitator superfamily domain, general substrate transporter
AALTg4714	2.A.1	2.52	6.00E-09	MFS general substrate transporter
AALTg6207	2.A.1	2.46	2.38E-07	MFS multidrug transporter
AALTg3946	2.A.1	2.36	2.63E-22	fructose facilitator
AALTg2291	2.A.1	2.25	1.34E-07	high-affinity nicotinic acid transporter
AALTg7156	2.A.1	2.21	6.56E-14	mfs transporter
AALTg2599	2.A.1	2.19	5.85E-06	pantothenate transporter
AALTg10470	2.A.1	2.05	4.61E-05	MFS multidrug transporter
AALTg9286	2.A.1	2.01	5.02E-03	retrograde regulation 2
AALTg10961	2.A.1	1.96	9.28E-08	MFS transporter
AALTg8610	2.A.1	1.82	2.30E-09	MFS gliotoxin efflux transporter
AALTg2816	2.A.1	1.82	3.62E-14	major facilitator superfamily transporter
AALTg5813	2.A.1	1.77	3.00E-05	MFS general substrate transporter
AALTg4424	2.A.1	1.46	4.86E-07	major facilitator superfamily transporter
AALTg3543	2.A.1	1.46	1.68E-05	MFS general substrate transporter

AALTg2466	2.A.1	1.46	2.53E-07	sugar transporter STL1
AALTg11845	2.A.1	1.43	2.24E-04	MFS monocarboxylate transporter
AALTg9974	2.A.1	1.40	4.09E-03	sugar transporter family
AALTg1811	2.A.1	1.20	3.19E-04	Major facilitator superfamily domain general substrate transporter
AALTg9261	2.A.1	-1.14	3.87E-04	sugar transporter
AALTg334	2.A.1	-1.23	1.84E-04	siderophore iron transporter mirB
AALTg6793	2.A.1	-1.55	8.26E-10	high affinity glucose transporter RGT2
AALTg7487	2.A.1	-1.61	2.21E-09	MFS transporter
AALTg3424	2.A.1	-1.65	4.85E-06	glucose transporter rco-3
AALTg5328	2.A.1	-1.76	8.50E-08	high affinity glucose transporter RGT2
AALTg5937	2.A.1	-1.88	6.46E-12	MFS monosaccharide transporter,
AALTg7371	2.A.1	-1.98	1.51E-13	MFS multidrug transporter
AALTg9765	2.A.1	-1.98	6.27E-05	major facilitator superfamily transporter
AALTg149	2.A.1	-2.31	9.82E-05	facilitated glucose transporter,
AALTg1579	2.A.1	-2.43	2.03E-06	nitrate transporter crnA
AALTg8701	2.A.1	-2.82	6.75E-28	hexose transporter
AALTg2286	2.A.1	-2.88	6.55E-05	glucose galactose transporter
AALTg10971	2.A.1	-3.22	2.32E-40	MFS multidrug transporter
AALTg8788	3.A.1	2.02	2.95E-15	cytosolic Fe-S cluster assembly factor NBP35
AALTg8644	3.A.1	1.20	1.55E-04	ATP-dependent permease MDL2
AALTg7483	3.A.1	3.53	2.61E-40	ABC transporter
AALTg6923	3.A.1	1.32	2.87E-06	ATP-binding cassette glutathione S-conjugate transporter
AALTg6773	3.A.1	1.72	4.46E-06	abc transporter
AALTg2358	3.A.1	1.22	4.46E-05	ABC transporter
AALTg2261	3.A.1	2.35	2.76E-18	cytosolic Fe-S cluster assembling factor cfd1
AALTg2410	3.A.1	-1.36	3.25E-04	ABC transporter

Supplementary Table S12 Differentially expressed genes encoding cytochrome P450s.

ID	log2FC	padj	annotation
AALTg473	3.27	7.08E-04	cytochrome P450
AALTg9998	3.23	2.83E-06	hypothetical protein W97_01942
AALTg5137	3.16	1.84E-14	cytochrome P450 4A10
AALTg3134	2.19	5.98E-08	pisatin demethylase
AALTg8160	1.21	7.97E-03	benzoate 4-monooxygenase cytochrome P450
AALTg366	1.10	2.46E-03	cytochrome P450
AALTg11894	-2.09	5.54E-05	bifunctional P-450:NADPH-P450 reductase
AALTg9357	-1.75	7.79E-04	phenylacetate hydroxylase
AALTg3330	-1.32	5.41E-03	benzoate 4-monooxygenase cytochrome P450,
AALTg7699	-1.31	1.45E-07	14-alpha sterol demethylase Cyp51B
AALTg7358	-1.30	1.80E-06	Cytochrome P450
AALTg8358	-1.16	2.41E-03	benzoate 4-monooxygenase cytochrome P450
AALTg7620	-1.07	1.50E-03	Cytochrome P450

Supplementary Table S13 Differentially expressed genes encoding heat shock proteins and ubiquitination related enzymes.

ID	log2FC	padj	annotation
AALTg1212	-2.06	7.75E-16	heat shock 90
AALTg4160	-1.03	2.11E-05	heat shock Hsp88
AALTg789	-1.85	2.42E-12	heat shock 70
AALTg4905	1.11	6.81E-04	DNAJ domain containing
AALTg10836	2.96	3.24E-33	DNAJ domain containing
AALTg2843	1.72	4.70E-10	Heat shock
AALTg5344	1.47	3.03E-05	Fe-S assembly co-chaperone
AALTg5883	1.15	3.44E-04	DnaJ domain containing protein
AALTg6765	-2.73	3.79E-35	DNAJ heat shock family
AALTg11445	-1.81	1.59E-14	chaperone dnaJ
AALTg3254	-1.31	6.20E-06	DNAJ domain containing
AALTg3033	-1.20	2.99E-06	DNAJ domain containing
AALTg10536	-2.06	2.69E-12	30 kDa heat shock
AALTg4088	4.23	3.43E-12	chaperone heat shock Hsp12
AALTg5164	4.00	7.13E-08	chaperone heat shock Hsp12,
AALTg6960	1.76	3.81E-03	ubiquitin-conjugating enzyme E2 2-like
AALTg4835	1.47	2.71E-09	ubiquitin-conjugating enzyme
AALTg10966	1.76	1.86E-13	ubiquitin carboxyl-terminal hydrolase 14

Supplementary Table S14 Primers used in qRT-PCR Analysis.

ID	Sequence
AALTg2809-F	ATTGGTAGTGGAGGTATTGC
AALTg2809-R	GTATGCCTTTGCGTCTTT
AALTg6701-F	CTCCTTCCCGCCTCTAC
AALTg6701-R	CTGTCGTCGCCAATCGT
AALTg10096-F	TTTTCGGCACCACTTTC
AALTg10096-R	TGCTTCAGGAGCTTCAACT
AALTg9320-F	ACCCAGCCACCTTTGAG
AALTg9320-R	TAAGCCTGCGTCGTCCT
AALTg3740-F	TGCCTGGAACCTTTACCT
AALTg3740-R	TCCGACAAGACCACCTACT
AALTg912-F	CACTCGCTGGAGGCTTTA
AALTg912-R	TGTTGTGCCGACTTGACG
AALTg8622-F	GGACGCCATGCTGAACC
AALTg8622-R	TGGAACCGACGGGATAA
AALTg10470-F	TCGCATTATACGCCTCCT
AALTg10470-R	ACGAACGACCCGACCAT
AALTg8644-F	AACGGAGTCATTTCTATCGG
AALTg8644-R	CTGGCAGACCCTTGGTG
AALTg2843-F	ATCTGGTGGCTATGGACG
AALTg2843-R	GGAACTTGATTGCCTTGCT
AALTg5164-F	TTCCGCAAAGATACCCA
AALTg5164-R	GTGACCCATTCCAAGAGC
AALTg9933-F	GACAAGGTCGCTTACAACG
AALTg9933-R	CACCACCAGGAACGAAAA
AALTg3416-F	TCGCACCCATTTACAACC
AALTg3416-R	CCCAACTCCGCAGCAAC
AALTg41-F	CAGGAGTTCTGCCAGATTAA
AALTg41-R	TCAGGCTTCTTGGTGCTT
AALTg4186-F	CGTTCTCCGTCGTTCCC
AALTg4186-R	TTCCACCATCAATGAGTTCTT
AALTg8569-F	ACACCACCCACGCAATC
AALTg8569-R	GCCGCATAACTCAGTCCTA
AALTg177-F	CAGCCATACCAAGTCCCG
AALTg177-R	CAGGCGTCCAGAGCAGA
AALTg11894-F	CCGCAGTGGGTATGACG
AALTg11894-R	GGCAAGCACCAAGAGGG
actin-F	GCCTTCCGTCTTGGGTCT
actin-R	AGGGCGGTGATTTCCTT
